# Pancreatic Cancer Organoids for Determining Sensitivity to Bromodomain and Extra-Terminal Inhibitors (BETi)

**DOI:** 10.3389/fonc.2019.00475

**Published:** 2019-06-05

**Authors:** Benjamin Bian, Natalia Anahi Juiz, Odile Gayet, Martin Bigonnet, Nicolas Brandone, Julie Roques, Jérôme Cros, Nenghui Wang, Nelson Dusetti, Juan Iovanna

**Affiliations:** ^1^Centre de Recherche en Cancérologie de Marseille (CRCM), INSERM U1068, CNRS UMR 7258, Aix-Marseille Université and Institut Paoli-Calmettes, Parc Scientifique et Technologique de Luminy, Marseille, France; ^2^Pathology Department, Beaujon Hospital, Assistance Publique-Hôpitaux de Paris, UMR 1149, Inflammation Research Center, INSERM - Paris Diderot University, Paris, France; ^3^Ningbo Wenda Pharma Technology Ltd., Zhejiang, China

**Keywords:** pancreatic cancer, organoids, c-MYC, NHWD-870, JQ1

## Abstract

Pancreatic ductal adenocarcinoma (PDAC) is a heterogeneous disease, therefore stratification of patients is essential to predict their responses to therapies and to choose the best treatment. PDAC-derived organoids were produced from PDTX and Endoscopic Ultrasound-Guided Fine-Needle Aspiration (EUS-FNA) biopsies. A signature based on 16 genes targets of the c-MYC oncogene was applied to classify samples into two sub-groups with distinctive phenotypes named MYC-high and MYC-low. The analysis of 9 PDTXs and the corresponding derived organoids revealed that this signature which was previously designed from PDTX is transferable to the organoid model. Primary organoids from 24 PDAC patients were treated with NHWD-870 or JQ1, two inhibitors of c-MYC transcription. Notably, the comparison of their effect between the two sub-groups showed that both compounds are more efficient in MYC-high than in MYC-low samples, being NHWD-870 the more potent treatment. In conclusion, this study shows that the molecular signatures could be applied to organoids obtained directly from PDAC patients to predict the treatment response and could help to take the more appropriate therapeutic decision for each patient in a clinical timeframe.

## Introduction

Pancreatic ductal adenocarcinoma (PDAC) is the 12th most commonly occurring cancer in men and the 11th in women. There were 460,000 new cases in 2018 with approximately the same number of deaths (1). Unfortunately, during the next decade this disease is projected to reach the second leading cause in terms of cancer related mortality in the western world (1). Like others malignant diseases, PDAC results from a complex combination of genetic, epigenetic, and environmental factors which gives rise to an heterogeneous disease, with patients having different set of symptoms, predisposition to early metastasis and therapeutic responses (2–4). This heterogeneity highlights the necessity to stratify patients with the goal of predicting better their responses to therapies in order to choose the best treatment for them (5–7).

One promising approach could be to determine novel therapy response biomarkers focused on the pathways that are critical for tumor growth and progression. In this context, the transcription factor c-MYC drives the expression of up to 1,500 genes involved in proliferation, cell metabolism, and apoptosis (8–10). Actually, this oncogene is implicated in the pathogenesis of one-third of all human malignancies and concerning PDAC etiology, c-MYC was found amplified in more than 30% of them (11). All of these features suggest that c-MYC behaves as a cancer driver gene in PDAC. In consequence, many efforts have been done to identify strong and specific MYC inhibitors. Key to this effort has been the discovery of Bromodomain and Extra-Terminal proteins inhibitors (BETi) which show an efficient inhibition of c-MYC by reducing its transcription level through BRD2/3/4 inhibition (12, 13). We recently described, in a cohort of 55 Patient-derived tumor xenografts (PDTX) from PDAC patients two subgroups of PDAC patients, named MYC-high and MYC-low (14). Although no significant differences on the c-MYC expression levels were found between both subgroups, a set of 16 transcriptional targets of c-MYC were able to define a molecular signature of its activity. Approximatively 30% of samples share a dependency toward c-MYC oncogene activity by overexpressing those genes. We also demonstrated that the MYC-high subgroup is sensitive to the canonical bromodomain inhibitor JQ-1 treatment. This increased sensitivity is mediated by a combination of cell cycle arrest followed by an increase in apoptosis rate, in several *in vivo* and *in vitro* PDAC models like PDTX, 2D cell monolayer and 3D aggregates (14).

Currently, several pre-clinical models are used to test drug responses, for example, PDTX which recapitulate very well the genetics and phenotypic events occurring in patient's tumor. Furthermore, the tumor-stroma crosstalk and the intra/inter-tumoral heterogeneity are also maintained in PDTX (15). However, the time required to generate enough biological material from PDTX, to test their sensitivity to anticancer drugs, is not compatible with the survival time of the PDAC patients since it still takes from 6 to 8 months. Therefore, another alternative approach could be to use “organoids” directly derived from patient's tumors. The organoids represent mini-avatars that can be 3D cultured *in vitro* from a wide range of cancers (e.g., small intestine, colon, kidney, stomach, liver, and pancreas). Like PDTX, organoids can recapitulate the combination of genetic events that occur in the patient's tumor. Organoids, contrary to PDTX, need only 2 or 3 weeks to produce enough material. This fact highlights the advantage to use this type of model in order to give accurate and rapid tumor pharmacotyping data.

In the present study, PDAC-derived organoids were produced from PDTX, on one hand, and directly from EUS-FNA biopsy, on the other hand. We showed that the 16-genes MYC targets related signature previously designed from PDTX is accurately transferable to organoids using the Nanostring custom codeset technology. And thus, it allows a rapid selection of MYC-high organoids for BETi treatments. Indeed, the MYC-high and MYC-low organoids samples were treated with the classical JQ-1 and with a novel BETi (NHWD-870) and as expected, MYC-high organoids are more sensitive to these treatments than MYC-low organoids.

## Materials and Methods

### PDTX and Organoids Samples

Patients were included under the Paoli Calmettes Institute clinical trial NCT01692873 (https://clinicaltrials.gov/show/NCT01692873). Consent's forms of informed patients were collected and registered in a central database. Two types of samples were obtained, namely Endoscopic Ultrasound-Guided Fine-Needle Aspiration (EUS-FNA) biopsies from patients with unresecable tumors, and tumor tissues from patients undergoing surgery (16). The percentage of EUS-FNA samples that we successfully culture as organoid is around 85%. PDAC samples were mixed with 100 μl of Matrigel (BD Biosciences) and implanted with a trocar (10 Gauge, Innovative Research of America, Sarasota, FL) in the subcutaneous right upper flank of an anesthetized and disinfected mouse. When tumors reached 1 cm^3^, mice were sacrificed and tumors were removed. All protocols in mice were carried out in accordance with the nationally approved guidelines for the treatment of laboratory animals. All procedures on animals were approved by the Comité d'éthique de Marseille numéro 14 (C2EA -14).

To obtain organoids from PDTX, xenografts were splited into several small pieces and processed in a biosafety chamber and after a fine mincing, they were treated with the Tumor Dissociation Kit (Miltenyi biotec). Undigested pellets were digested with accutase (Sigma) at 37°C for 30 min. The pancreatic tissue slurry was transferred into a tissue strainer 100 μm and were placed into 12-well plate coated with 150 μl GFR matrigel (Corning). The samples cultured with Pancreatic Organoid Feeding Media (POFM) consisted of Advanced DMEM/F12 supplemented with 10 mM HEPES (Thermo-Fisher); 1x Glutamax (Thermo-Fisher); penicillin/streptomycin (Thermo-Fisher); 100 ng/ml Animal-Free Recombinant Human FGF10 (Peprotech); 50 ng/ml Animal-Free Recombinant Human EGF (Peprotech); 100 ng/ml Recombinant Human Noggin (Biotechne); Wnt3a-conditioned medium (30% v/v); RSPO1-conditioned medium (10% v/v); 10 nM human Gastrin 1 (Sigma Aldrich) 10 mM Nicotinamide (Sigma Aldrich); 1.25 mM N acetylcysteine (Sigma Aldrich); 1x B27 (Invitrogen); 500 nM A83-01 (Tocris); 10.5 μM Y27632 (Tocris). The plates were incubated at 37 °C in a 5% CO_2_ incubator, and the media were changed every 3 or 4 days.

Finally, primary PDAC-derived organoids were obtained from patients with unresecable tumors by EUS-FNA. Biopsy was digested rapidly with Dissociation Kit at 37°C for 5 min and was incubated with Red Blood Cell Lysis Buffer (Roche) and washed 2 times with PBS. The samples were then cultured as described above.

### Immunohistochemistry

Organoids and PDTX were Hematoxilin and Eosin stained. Immunoflourescence staining with COL-IV and ZO-1 antibodies was performed using the anti-Collagen IV rabbit polyclonal (Abcam ref ab6586) and the anti-ZO1 tight junction protein monoclonal antibody (ThermoFisher ref Z01-1A12) antibodies following standard methods.

### Gene Expression Quantification and MYC Signature Scoring

Sixteen genes were selected known to be over-expressed (CAD, CCT4, CDC20, KPNA2, MAD2L1, MCM2, PLK1, RFC4, RUVBL2, and SRM) or under-expressed (BCL2L15, CTSE, ERN2, RAB25, TXNIP, and VSIG2) by MYC as the MYC signature already defined by our group (14). Probe sets for each gene were designed and synthesized by NanoString technologies (Table 1). Probe sets of 100 bp in length were designed to hybridize specifically to each mRNA target. Probes contained one capture probe linked to biotin and one reporter probe attached to a color-coded molecular tag, according to the Nanostring code-set design. We used 100 ng of total RNA isolated from each organoid as suggested by the manufacturer. Technical replicates of samples were included. Data were analyzed using the nCounter™ digital analyzer software, available at http://www.nanostring.com/support/ncounter/.

**Table 1:** Sixteen genes used for determine the MYC signature and 4 genes used as housekeeping reference.

**MYC signature**		
**Gene name**	**Accession number**	**NanoString probe ID**
BCL2L15	NM_001010922.2	NM_001010922.2:3363
CAD	NM_004341.3	NM_004341.3:2380
CCT4	NM_006430.3	NM_006430.3:1191
CDC20	NM_001255.2	NM_001255.2:430
CTSE	NM_001910.2	NM_001910.2:2070
ERN2	NM_033266.3	NM_033266.3:1132
KPNA2	NM_002266.2	NM_002266.2:917
MAD2L1	NM_002358.3	NM_002358.3:668
MCM2	NM_004526.3	NM_004526.3:1296
PLK1	NM_005030.3	NM_005030.3:535
RAB25	NM_020387.3	NM_020387.3:668
RFC4	NM_181573.2	NM_181573.2:1035
RUVBL2	NM_006666.1	NM_006666.1:369
SRM	NM_003132.2	NM_003132.2:512
TXNIP	NM_006472.3	NM_006472.3:2626
VSIG2	NM_014312.3	NM_014312.3:797
**HOUSEKEEPING GENES**		
SDHA	NM_004168.4	NM_004168.1:230
CLTC	NM_004859.3	NM_004859.2:290
TBP	NM_003194.5	NM_001172085.1:587
GUSB	NM_000181.4	NM_000181.3:1899
RPL19	NM_000981.4	NM_000981.3:315

Heatmaps were generated by hierarchical clustering analysis on GENE-E software (version 3.0.204; Broad Institute, Cambridge, MA, USA). Sixteen differentially expressed genes between the MYC-high and MYC-low clusters were represented.

In order to calculate the MYC signature scoring, 24 consecutive PDAC samples directly from EUS-FNA biopsies were obtained and cultivated them for 2–3 weeks as organoids before RNA extraction. RNAs were analyzed by the Nanostring codeset with the 16 MYC-associated transcripts. Data was analyzed as previously described (14). Briefly, when a transcript was over-expressed as expected in MYC-high samples, the up/down ratio should be >1. Conversely, in samples from patients with a MYC-low activity this ratio should correspond to < 1. Normalization was calculated as follows: first, the sum of the expression values of all patients of each up-regulated gene (e.g., for gene a: P1a + P2a + P3a + …P24a) was given the arbitrary value of 100. In the same way, the expression of down-regulated genes was normalized (e.g., for gene A: P1A + P2A + P3A + …P24A = 100). After that normalization, for each patient the ratio between each up-regulated and down-regulated transcript was calculated as follows: a/A, a/B, a/C, a/N; b/A, b/B, b/C, b/N, etc. When the median of these ratios were over 1, the PDAC samples were considered as MYC-high, whereas when the median was < 1, the sample was assigned to the MYC-low profile.

### Chemograms

Organoids were screened for chemosensitivity to two BETi: JQ1 (12) and NHWD-870 (gift from Ningbo Wenda Pharma Technology LTD, Zhejiang, China). Twenty four primary organoids were treated for 72 h with increasing concentrations of BETi drugs ranging from 0 to 100 μM. Each experiment was performed in triplicate and repeated at least two times. Cell viability was estimated after addition of the PrestoBlue cell viability reagent (Life Technologies) for 3 h following the protocol provided by the supplier.

### Statistical Analysis

The IC50 and AUC values were calculated from a log (drug) vs. normalized response curve with robust fit using GraphPad Prism software v5.0 (GraphPad Software). Data for cell viability assays were analyzed using one-way repeated analysis of variance (ANOVA) with Dunnett *post-hoc* test for multiple comparisons. A *p*-value < 0.05 is considered significative.

## Results

### Phenotype Characterization and Comparison Between PDAC-Derived PDTX and Organoids Models

**Figure 1:**
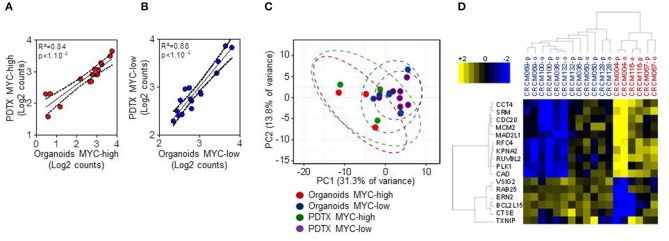
Nanostring code set for PDAC MYC-high and MYC-low subgroups assignment in 9 patients with PDTX and their corresponding organoids. **(A,B)** Pearson correlation analysis of the 16-genes signature between PDTX and organoids. The expression of each gene was averaged in log2 counts scale within each sub-group in each model (MYC-high samples in **(A)** (red dots) and MYC-low samples in **(B)** (blue dots). **(C)** Principal Component Analysis (PCA) using the 16 Nanostring codeset (red dots: MYC-high organoids; green dots: MYC-high PDTX; blue dots: MYC-low organoids; and purple dots: MYC-low PDTX). **(D)** Heatmap showing the expression of the 16-genes signature along 6 MYC-low and 3 MYC-high patients. Blue scale indicates under-expressed genes and yellow scale up-regulated genes. -o, organoids; -p, PDTX.

We first studied the maintenance of the phenotype of organoids when conserved in culture. With this aim, we derived organoids from PDTX obtained from PDAC tumors and compared the phenotype of both models by morphologic and transcriptomic criteria. First, we analyzed the expression levels of the 16-genes that we had previously defined by microarray assay as being associated to the MYC signature (14). In the current study, instead of a transcriptomic approach we performed a Nanostring codeset on those genes for 9 PDTX and their corresponding derived organoids. The expression of each gene was averaged in log2 counts scale and it allowed us to classify samples into the two sub-groups named MYC-high (*n* = 3) and MYC-low (*n* = 6). Figures 1A,B present the Pearson correlation analysis of the 16-genes signature between PDTX and their organoids. Using these data we also performed a Principal Component Analysis (PCA). Figure 1C represents the distribution of the RNA expression profiles of MYC-high organoids (red dots) and PDTX (green dots), and MYC-low organoids (blue dots) and PDTX (purple dots). This analysis shows that the distance between each dot is related to the similarity between observations in high-dimensional space. From these data we may assume that expression of the genes associated to the MYC-signature is strongly conserved between PDTX and their derived organoids. Finally, we represented the transcriptome of the 16 MYC-associated genes on a heatmap presented in Figure 1D. We can observe, on one hand, that MYC-high and MYC-low phenotypes are clearly separated and, on the other hand, that PDTX and organoids are generally associated with only few exceptions.

**Figure 2:**
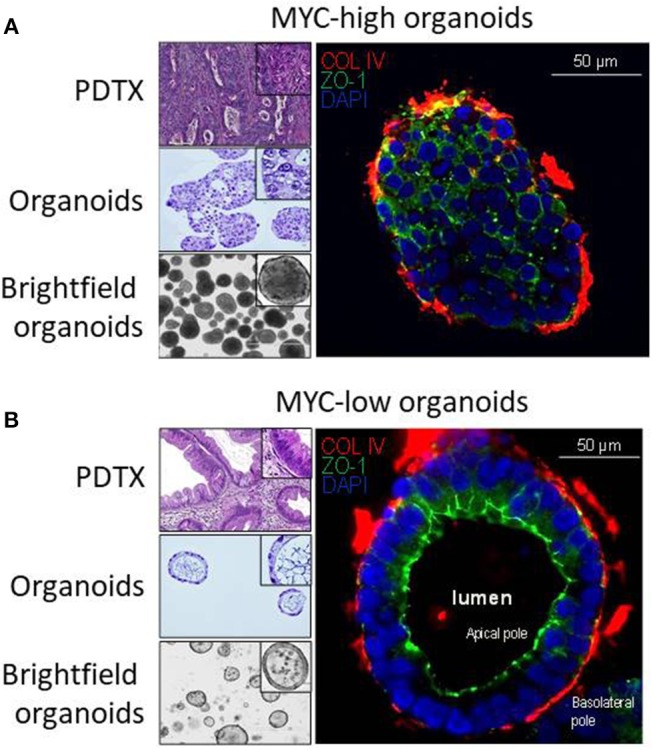
Characterization of PDAC-derived PDTX and organoids models. Characterization of the macrostructures in MYC-high and MYC-low subgroups by H&E and immunofluorescence staining. **(A)** MYC-high PDTX and organoids H&E staining (top-left and middle-left, respectively) show no gland structures. The corresponding organoids are organized as a full structure reminding cellular 3D aggregates with low polarization. **(B)** MYC-low subgroup is characterized by well-organized gland structures (upper-left) with columnar epithelium. Organoids show a majority of regular cystic structures composed by an apical and basolateral compartment. ZO-1, marker for apical membrane, is in green and COL-IV, marker for base membrane, is in red. Scale bar is 50 μm.

Then, we analyzed the correlation between the gene expression profile and the morphology. Figures 2A,B show representative pictures of PDTX and organoids from MYC-high and MYC-low phenotype, respectively. MYC-high PDTX phenotype shows few glandular structures, it is poorly colonized by stroma and shows a high nucleocytoplasmic ratio; whereas the MYC-low PDTX subgroup presents glandular structures with a columnar epithelium and abundant lumen. Organoids derived from MYC-high PDTX grown as a compact structure with no signs of cellular differentiation and polarization and with scarce or in some cases absence of lumen. On the contrary, the cells of the organoids derived from MYC-low PDTX are well-polarized to form glandular structures and these glands show the presence of abundant lumen. Altogether, these data indicate that phenotype and transcriptome of the MYC-associated genes in organoids derived from PDTX are well-conserved suggesting that organoids could be a putative source of RNA for detecting PDAC sensitives to the chemotherapies.

### MYC Signature Scoring Primary Organoids From 24 PDAC Patients

**Figure 3:**
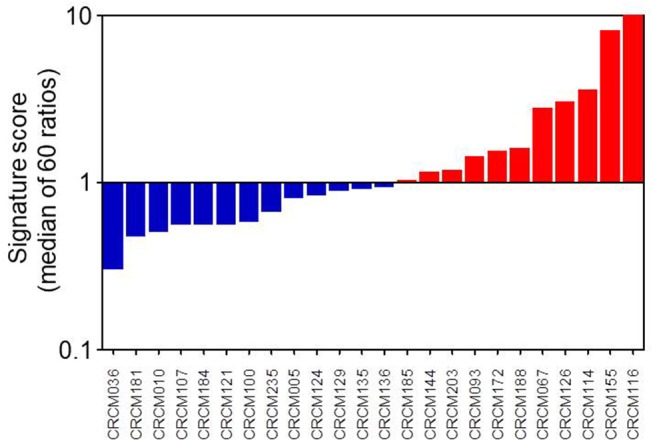
MYC signature scoring 24 primary PDAC-derived organoids. As described in M&M section, when a transcript was activated as expected in MYC-high samples, the up/down ratio should be > 1. Conversely, in samples from patients with a MYC-low activity it is predicted a ratio <1. After normalization of all 16 transcripts, we considered that organoids corresponded to a MYC-high profile when the median of these ratios was over 1 and to a MYC-low profile when the median was <1.

We obtained 24 consecutive PDAC samples directly from EUS-FNA biopsies and cultivated them for 2–3 weeks as organoids before RNA extraction. These RNAs were analyzed by the Nanostring codeset with the 16 MYC-associated transcripts. After score calculation, we were able to classify the 24 samples into 11 putative MYC-high (45.8%) and 13 putative MYC-low (54.2%) according to their signature scores, as presented in Figure 3. We also performed the analysis of the score of our signature on already published data sets. When calculating this score for the ICGC cohort (dcc.icgc.org, release 20) (*N* = 269), which transcriptome was obtained by microarray, the percentages MYC-high and MYC-low were 49.1 and 50.9%, respectively. In the RNA-Seq TCGA cohort (gdac.broadinstitute.org) (*N* = 150) 44.3% of patients were classified as MYC-low, whereas 55.7% as MYC-high.

### Effect of BETi Treatment on MYC-High and MYC-Low Organoids

**Figure 4:**
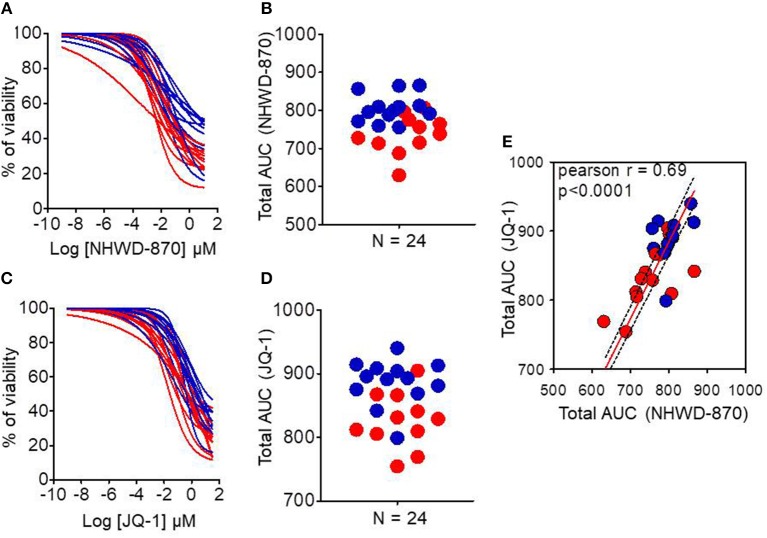
Chemograms on MYC-high and MYC-low organoids upon BETi treatments. Dose-response curves (chemograms) and total Area Under the Curve (AUC) distribution for JQ-1 **(A,B)** and NHWD-870 **(C,D)**. **(E)** Correlation between total AUC for JQ-1 (y-axis) and NHWD-870 (x-axis). The red curves and dots represent MYC-high and blue curves and dots represent MYC-low organoids. Drugs concentrations were Log-transformed μmol/l. *n* = 24 in each case.

PDAC-derived organoids were treated with increasing doses of two different BET proteins inhibitors named JQ-1 and NHWD-870 to verify that the MYC signature obtained directly on organoids from patients was able to predict their sensitivity. Figures 4A,C show the dose response curves of these organoids for JQ-1 or NHWD-870 compounds, respectively. Figures 4B,D represent the area under the curve (AUC) corresponding to each organoid of JQ-1 and NHWD-870, respectively. The AUC for the treatment with JQ-1 of MYC-high (*n* = 11) is 826.4 ± 43.6 whereas for MYC-low (*n* = 13) is 886.6 ± 35.8 (*p* = 0.0026). Similarly, the AUC for the NHWD-870 treatment of MYC-high is 737.6 ± 51.1 and 806.2 ± 36.7 for MYC-low (*p* = 0.0026). Importantly, the AUCs of organoids treated with NHWD-870 are significantly lower compared to JQ-1 indicating that NHWD-870 is a most potent BETi compound. In Figure 4E it is showed the correlation between total AUC for JQ-1 in y-axis and NHWD-870 in x-axis. On one hand, we observe a significant correlation in the responses to both compounds (*p* < 0.0001) and, on the other hand, that the AUCs for the MYC-high organoids are significantly lower than MYC-low organoids.

## Discussion

In this work we demonstrated that PDAC-derived organoids conserve the structure of the deriving tissue, at least in terms of morphology and the expression levels of the genes involved in the MYC signature (14). This suggests that these tissues could be used as a source of biological material for selecting a more adapted treatment for each patient with PDAC. In a previous study, we proposed a strategy for rapid selection of personalized treatments for PDAC patients which consisted in defining their sensitivity by transcriptomic characterization rather than pharmacological tests (16, 17). In fact, using PDTX for prediction of the response to the treatment is a good approach (18). However, it is incompatible, or at least not systematically applicable, due to the short time of survival expected for patients with a PDAC. The best way to obtain a transcriptome profile would be directly from EUS-FNA that can be taken from non-resecable and metastasic tumors, it has still big limitations. In one hand, the contamination with blood and stroma cells make impossible to assign an adequate pathology report by bulk sequencing. Moreover, although this material is less exposed to digestion by RNAses than normal pancreatic tissue, degradation remains very high to analyze RNA of quality (19). Therefore, PDAC-derived organoids are *in vitro* tumor models, derived from surgical or EUS-FNA specimens, that serves as source of pure and high-quality material. In this way, they could be used not only as a tool for defining molecular signature but also to personalize treatments in a clinical timeframe. Creation of human PDAC organoids at the time of initial tumor diagnosis is therefore critical. Our aim was to assess the feasibility of creating a human PDAC organoids cohort obtained by EUS-FNA sampling in patients with non-operable PDAC. Some recent works focuses on organoids as a tool to analysis the drug screening toward personalized medicine in PDAC (20–22).

In this paper we demonstrated, as a proof of concept, that the use of PDAC-derived organoids from EUS-FNA specimens is a promising strategy to obtain clean transformed material and can help to take the better decision for a particular patient. However, contrary to the previously published strategies which tried to perform chemograms on these organoids to define the sensitivity to a given drug, our approach consists in the analysis of the phenotype by selecting a set of RNAs associated to the sensitivity to one drug applying a transcriptional signature. In this way, gene expression could be used for testing signatures for different drugs from the same dataset, reducing the necessary starting material. In this study, we demonstrated that using our already stablished signature (14) we were able to classify tumors with high MYC activity and therefore predicted to be more sensitive to BETi in a short time such as 2–3 weeks after obtaining the samples from the patients.

Interestingly, organoids defined as high MYC activity present a different phenotype from that with low MYC activity, the last being characterized by a most differentiated morphology as presented in Figure 2. This is in agreement with our previous data showing that PDTX with high MYC activity present a basal rather than a classical phenotype (14). These results may be indicating that MYC activity could be essential in establishing the PDAC phenotypes.

In PDAC, epigenetic landscape is intensely studied for therapeutic drug discovery, showing great promise as recently reported (23). JQ1 has proven to be a first-in-class, drug-like inhibitor of the “Bromodomain and Extraterminal Domain” epigenetic readers (BETs), which recognize histone lysine acetylation marks. JQ1 has facilitated the mechanistic study and therapeutic application in cancer of this kind of epigenetic inhibition. For example, it has been already shown its tumor growth suppression capacity in PDAC patient-derived xenograft models (24). This drug down-regulates the MYC transcriptional program since BET activity is necessary for its transcriptional function (25). However, drugs with the same therapeutic targets can have different toxicity and pharmacological results because of their on-target and off-target toxicities and pharmacokinetics, resulting in different clinical applications. Therefore, new BETi with different chemotypes are being studied in order to exploit the whole therapeutic potential of the BET inhibition. NHWD-870 is a novel, potent and selective BET family bromodomain inhibitor that only binds to bromodomains of BRD2/3/4/T. NHWD-870 is a carboline derivate compound that has a powerful tumor suppressive efficiency in xenograft mouse models of small cell lung cancer, triple negative breast cancer and ovarian cancer (26). Moreover, a recent article has provided as proof-of-concept the combination of NHWD-870 with agents targeting the IGF1R pathway for treating advanced Ewing sarcoma (27). These results support its further development for diverse solid tumor indications in clinic including PDAC. We selected these compounds to compare their activity in both MYC-high and MYC-low organoids and found that both compounds are more efficient in MYC-high than in MYC-low and that NHWD-870 is more potent than JQ1 (Figure 4).

In conclusion, in this work we have demonstrated that using organoids obtained directly from PDAC patients could be used to predict the response to chemotherapy and help to decide for the more appropriate therapeutic decision for each patient.

## Data Availability

Publicly available datasets were analyzed in this study. This data can be found here: http://gdac.broadinstitute.org/; https://dcc.icgc.org/

## Ethics Statement

This study was carried out under the protocol ID RCB: 2011-A01439-32, Comité de Protection des Personnes Sud-Méditerranée I and tha Paoli Calmettes Institute clinical trial NCT01692873 (https://clinicaltrials.gov/show/NCT01692873). All subjects gave written informed consent in accordance with the Declaration of Helsinki.

All protocols in mice were carried out in accordance with the nationally approved guidelines for the treatment of laboratory animals. All procedures on animals were approved by the Comité d'éthique de Marseille numéro 14 (C2EA -14).

## Author Contributions

BB, OG, MB, NB, and JR performed the experiments. BB, JC, ND, and JI participated in the experimental design. NW provided material. BB, NJ, ND, and JI conceived, wrote the manuscript. JC, NJ, ND, and JI participated in the paper discussion.

### Conflict of Interest Statement

NW was employed by company Ningbo Wenda Pharma Technology Ltd., Zhejiang, China. The remaining authors declare that the research was conducted in the absence of any commercial or financial relationships that could be construed as a potential conflict of interest.
